# Introduction to the Special Issue: The role of seed dispersal in plant populations: perspectives and advances in a changing world

**DOI:** 10.1093/aobpla/plaa010

**Published:** 2020-03-13

**Authors:** Noelle G Beckman, Clare E Aslan, Haldre S Rogers

**Affiliations:** 1 Department of Biology and Ecology Center, Utah State University, Logan, UT, USA; 2 School of Earth and Sustainability, Northern Arizona University, Flagstaff, AZ, USA; 3 Department of Ecology, Evolution, and Organismal Biology, Iowa State University, Ames, IA, USA

**Keywords:** CoDisperse, defaunation, frugivores, plant recruitment, population dynamics, population spread, seed dispersal

## Abstract

Despite the importance of seed dispersal as a driving process behind plant community assembly, our understanding of the role of seed dispersal in plant population persistence and spread remains incomplete. As a result, our ability to predict the effects of global change on plant populations is hampered. We need to better understand the fundamental link between seed dispersal and population dynamics in order to make predictive generalizations across species and systems, to better understand plant community structure and function, and to make appropriate conservation and management responses related to seed dispersal. To tackle these important knowledge gaps, we established the CoDisperse Network and convened an interdisciplinary, NSF-sponsored Seed Dispersal Workshop in 2016, during which we explored the role of seed dispersal in plant population dynamics (NSF DEB Award # 1548194). In this Special Issue, we consider the current state of seed dispersal ecology and identify the following collaborative research needs: (i) the development of a mechanistic understanding of the movement process influencing dispersal of seeds; (ii) improved quantification of the relative influence of seed dispersal on plant fitness compared to processes occurring at other life history stages; (iii) an ability to scale from individual plants to ecosystems to quantify the influence of dispersal on ecosystem function; and (iv) the incorporation of seed dispersal ecology into conservation and management strategies.

## Teaser

Seed dispersal is fundamental to the structure and function of plant communities, but its complexity and heterogeneity impede mechanistic understanding and quantitative prediction of seed dispersal processes and their disruption. In this Special Issue, we consider the current state of seed dispersal ecology and identify collaborative research needs. The diversity of disciplines, geographic regions and expertise represented in this Special Issue yield a range of perspectives and insights and, we hope, will stimulate further collaborations to advance seed dispersal ecology and conservation.

## Introduction

**Figure 1.**
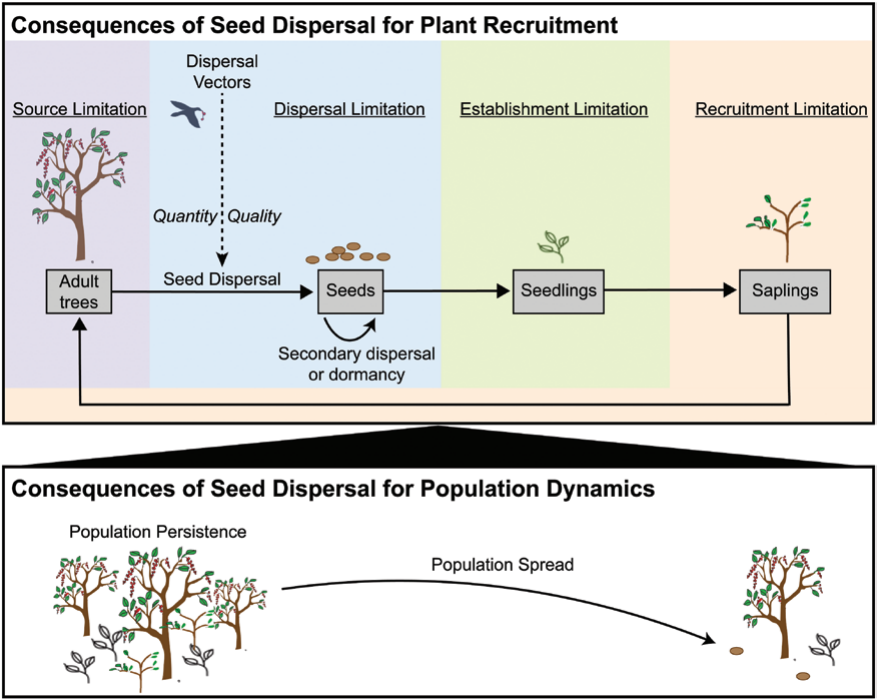
Consequences of seed dispersal for plant recruitment and population dynamics. Shadings indicate limitation imposed at different stages by the seedscape, the local environment surrounding a seed following seed dispersal that influences later stages of plant recruitment, such as conspecific distance and density, substrate and light (Beckman and Rogers 2013).

Dispersal is a central component of conceptual frameworks within ecology and evolution (Clobert *et al.* 2012; Vellend 2016). Movement of individuals affects the spread of populations (Cain *et al.* 2000), patterns of biodiversity across local, regional and global scales (Vellend 2010), genetic diversity and adaptive capacity (Kremer *et al.* 2012) and species’ responses to global change (Travis *et al.* 2013). For a plant, movement of the seed provides the single opportunity in its entire life cycle to change its geographic location. As the seed is deposited within the seedscape (Beckman and Rogers 2013), this life history event sets the template for all future interactions in a plant’s life (Fig. 1; Nathan and Muller-Landau 2000). Seed dispersal is of great conservation importance, since it is both affected by global change and affects the ability of plants to move or adapt to global change (McConkey *et al.* 2012; Travis *et al.* 2013). As such, seed dispersal holds the potential to affect patterns of biodiversity by mediating population- and community-level dynamics.

In spite of the importance of seed dispersal as a driving process behind plant community assembly, our understanding of the role of seed dispersal in plant population persistence and spread remains incomplete. As a result, our ability to predict the effects of global change on plant species populations is hampered. This lack of knowledge persists primarily because the processes underlying the dispersal of seeds, as well as their consequences, are complex and context-dependent (Fig. 1; Wang and Smith 2002; Beckman *et al.* 2019). *In order to make predictive generalizations across species and systems, to better understand plant community structure and function, and to make appropriate conservation and management decisions related to seed dispersal, we need to better understand the fundamental link between seed dispersal and population dynamics.* In this Special Issue, we propose interdisciplinary approaches to advance our understanding of the importance of seed dispersal for plant population dynamics.

## Insights from the Special Issue

**Figure 2.**
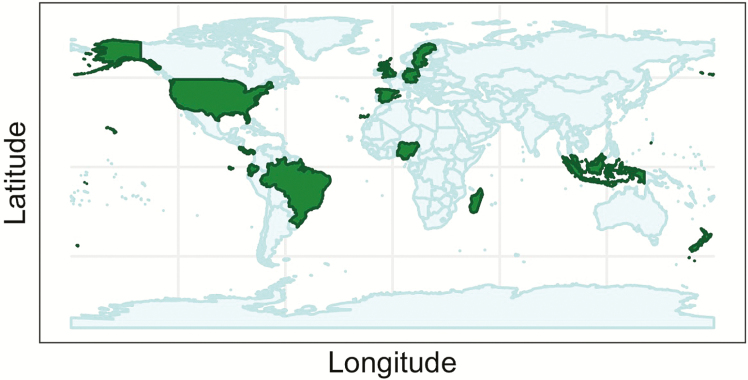
Map of countries. Participants were actively conducting research in 15 countries (shown in green).

Progress in basic research into seed dispersal ecology and its application in conservation and management has been limited by a lack of interactions among disciplines and biogeographical regions. Often individual researchers attend only disciplinary conferences and read disciplinary journals; therefore, researchers using mathematical biology approaches to model the dispersal process may not interact with ecologists empirically studying the dispersal process, for example. However, seed dispersal links disciplines and subdisciplines from physiological ecology to community ecology to biogeochemistry to computational and mathematical modeling. Geographical boundaries are also limiting, often with little interaction among researchers working in temperate vs. tropical systems and Old World vs. New World systems. Even the research focus on a dispersal mode produces boundaries, with little interaction between groups studying wind dispersal and vertebrate dispersal, for example. To identify and tackle knowledge gaps on the role of seed dispersal in plant population dynamics, we established the CoDisperse Network of researchers and convened an interdisciplinary, NSF-sponsored Seed Dispersal Workshop in 2016 (NSF DEB Award # 1548194). Thirty workshop participants came from six countries (Canada, Germany, Nigeria, Switzerland, UK and the USA) and were actively conducting research in 15 countries across North and South America, Europe, Africa, Asia and Oceania (Fig. 2). Participants brought a range of expertise including ecology, evolution, animal behaviour, conservation biology, environmental policy, math, theory, biogeography, molecular biology, genomics, physics, statistics and complex systems.

Using small- and large-group guided discussions designed to capture the varied perspectives of workshop participants, we explored the current state of seed dispersal ecology, specifically focusing on the consequences of seed dispersal for plant populations. We identified the following collaborative research needs: (i) the development of a mechanistic understanding of the movement process influencing dispersal of seeds; (ii) improved quantification of the relative influence of seed dispersal on plant fitness compared to processes occurring at other plant life history stages; (iii) an ability to scale from individual plants to ecosystems to quantify the influence of dispersal on ecosystem function; and (iv) the incorporation of seed dispersal ecology into conservation and management strategies. The workshop and papers in this Special Issue focus on strategies to advance the first three research needs, and we urge researchers and managers to continue discussions to advance the fourth research need.

To address these research needs, we argue that multiple epistemologies, or ways of knowing, are necessary and that discovering generality in patterns and processes requires comparing knowledge across systems. Overcoming epistemological barriers—disciplinary differences regarding research goals, theoretical frameworks, assumptions, rigor and causal explanation (Brister 2016)—to value multiple ways of knowing can lead to more integrative and innovative solutions to complex problems (Miller *et al.* 2008). We propose that seed dispersal is measurable and more predictable than traditionally assumed, and research advances and interdisciplinary collaborations are necessary to generalize across species and systems and make predictions in the context of global change. The reviews and viewpoints in this Special Issue provide specific recommendations for future research to make seed dispersal ecology generalizable and predictable utilizing interdisciplinary approaches, while the research papers give examples of applying novel empirical, mathematical, statistical and integrated approaches to address the identified knowledge gaps.

Aslan *et al.* (2019), Beckman *et al.* (2019) and Rogers *et al.* (2019) review our current standing in the field of seed dispersal ecology and propose interdisciplinary advances to approach the complexity of seed dispersal processes for generalization and prediction. Beckman *et al.* (2019) focus on promising interdisciplinary approaches for studying, generalizing and predicting seed dispersal and its demographic consequences. They begin with a general approach for studying the context dependency of seed dispersal, discuss strategies to reduce and embrace complexity, and encourage simultaneous and iterative data collection and model development. They consider advances and challenges associated with the first two aforementioned research needs, specifically (i) the mechanisms underlying the movement and resulting patterns of seeds and (ii) integrating seed dispersal with demography to examine the demographic consequences of this movement. Aslan *et al.* (2019) propose a functional approach to reducing the complexity of studying seed dispersal. They propose categorizing plants based on whether it matters (i) if seeds are dispersed, (ii) into what context they are dispersed and (iii) what vector disperses them. They explore the use of these functional group categories to achieve generalization across species and systems in seed dispersal ecology, a key challenge in the field. Rogers *et al.* (2019) revisit an existing but underutilized concept in seed dispersal—the total dispersal kernel. While most plants have multiple dispersal vectors, few studies incorporate the relative role of each vector on the overall dispersal kernel. Rogers *et al.* (2019) review empirical, theoretical and statistical challenges associated with studying the total dispersal kernel and suggest promising ways forward. Understanding the influence of each vector is critical as disperser communities are altered in today’s changing world.

Another key theme that emerged from the workshop was the importance of intraspecific variation in seed dispersal-related traits. Intraspecific variation can have important ecological and evolutionary consequences; however, most ecological models that investigate these consequences for population dynamics, species interactions and global change use mean values for species (Bolnick *et al.* 2011; Moran *et al.* 2016). Schupp *et al.* (2019) delve into the intrinsic and extrinsic drivers of intraspecific variation in seed dispersal. They find evidence that, while drivers of intraspecific variation in seed dispersal are diverse and pervasive, there are large gaps in our understanding, and they propose research to fill those gaps. Snell *et al.* (2019) synthesize research on the consequences of intraspecific variation in dispersal for population, communities, evolution and responses to global change. As an example of these consequences, Schreiber and Beckman (2020) use a mathematical approach to examine how intraspecific variation in dispersal and fecundity and their co-variation influence the spread rates of populations and apply this approach to a well-studied example, *Acer rubrum.* They find that population spread is highest when variation in dispersal co-varies positively with fecundity. Johnson *et al.* (2019) examine evidence for rapid changes in seed dispersal and the potential underlying mechanisms, including the role of phenotypic plasticity, epigenetics and rapid evolution. These papers highlight the importance of estimating intraspecific variation in dispersal and its drivers for predicting the persistence and spread of populations, community dynamics and coexistence.

One of the driving motivations behind the workshop was to understand how dispersal is influenced by global change drivers. Using an accidental experiment of previously logged, hunted and protected forests in the northern Republic of Congo, Nuñez *et al.* (2018) evaluate the effects of hunting and logging on tree fecundity and seed dispersal in the northern Republic of Congo. They find that low-intensity logging affected seed dispersal distances, though the direction and magnitude varied by species. Olsson *et al.* (2019) investigate how plant performance is affected by competition and reduced seed dispersal due to hunting and found that dispersal limitation was more important than competition for seedling recruitment in hunted forests. Both studies show that anthropogenic pressures influence seed dispersal. As the Earth continues to warm and overharvesting, logging and landscape fragmentation escalate, future work should continue to disentangle the effects of interacting pressures on seed dispersal and assess their implications for future plant communities.

## Interdisciplinary Collaboration to Advance Seed Dispersal Ecology

This Special Issue resulted from new collaborations across disciplines forged during the week-long Seed Dispersal Workshop. The workshop facilitated collaboration among empirical ecologists who conduct observational and experimental studies to examine the role of seed dispersal in plant ecosystems around the world; theoretical ecologists applying conceptual, statistical and mathematical models to generalize the importance of dispersal to plant populations across systems and species; and applied mathematicians, physicists and systems biologists identifying mathematical challenges and developing novel analytical tools, modeling approaches and efficient simulation platforms to deal with these multiscale problems.

There are many challenges and opportunities associated with bringing together a diverse group of experts for a short amount of time (Palmer *et al.* 2016). Having a diverse group of participants draws attention to the differences that exist across disciplines, ways of knowing and perspectives, including distinct language/jargon and disparate conceptualizations of the problem. We found that successful interdisciplinary interactions required a variety of approaches including defining discipline-specific jargon to provide a common language for researchers from all disciplines and collectively developing a framework for synthesis. Building on practices developed by the National Socio-Environmental Synthesis Center for interdisciplinary research (Palmer *et al.* 2016), we provide an overview of the approach we used for organizing and structuring an interdisciplinary workshop (Box 1) and more details in the **Supporting Information—File S1**. Throughout the Special Issue, we provide guidelines for researchers interested in crossing disciplinary boundaries.

**Box 1. Overview of Organizing an Interdisciplinary Workshop**

- In selecting the group, leaders must balance the breadth of representation of research areas while keeping the overall size to a manageable number for productive collaboration. Carefully select participants who offer diverse perspectives, are committed to achieving a common goal, and will do their part to create an open and welcoming environment.
- Select a location that has one large room and several breakout rooms, all with whiteboards and audio-visual capabilities.
- If IT support is available, virtual participants can be included, but we recommend carefully planning activities with virtual participants in mind in order to ensure seamless participation and positive group dynamics (Fraser *et al.* 2017).
- Start preparing and engaging participants early to set up an atmosphere of shared responsibility for the workshop’s success. Survey participants for advice on topics, strategies for organizing effective workshops, and shared outcomes and common goals.
- In a pre-workshop meeting, summarize results of pre-workshop surveys and provide expectations for workshop goals, objectives, outcomes, communication and participation before, during and after the workshop.
- Assign clear and differentiated roles and tasks to participants before, during and after the workshop.
- Keep focused. Set out a series of tractable questions that can be tackled in the period of time allotted. We suggest one overarching question and no more than 2–3 subquestions for the duration of a 1-week workshop. Visibly display these guiding questions in the central gathering place and revisit them often.
- Schedule the week carefully, making sure to allow time for team-building and open discussion, while also steering the group towards the final goals.
- Provide plenty of icebreakers that promote communication and expose differences and similarities in perspectives.
- Get everyone on the same page with regard to the state of the field from various perspectives with short overview presentations and panel discussions.
- Develop a shared vocabulary.
- Use teaching techniques designed to elicit contributions from all participants including small-group discussions, think-pair-share, brainstorming and sharing ideas anonymously on post-its, etc. Limit the use of large-group discussions to information sharing, initial brainstorming and report-backs, to reduce the likelihood of having a few voices dominate.
- Do not let discussions be derailed. Enforce this by requiring frequent report-backs from small groups using structured templates and providing a space for interesting ideas to be recorded and revisited later during the workshop or in future collaborations.
- Ensure interest/buy-in from all disciplines by allowing people to choose working groups of most interest and move to different working groups if they feel so inclined.
- Provide drinks, snacks and lunches to build group cohesion.
- Demonstrate strong, shared leadership before, during and after the workshop to create a respectful, open, inclusive and positive environment and to lead products to completion.
- Have groups identify next steps and select a leader for any projects that emerge from the workshop.
- Check in with project leaders and set deadlines for manuscripts. Conference symposia are useful for consolidating ideas for a review paper, and a Special Issue is a helpful tool for providing a single deadline for multiple papers emerging from a workshop.

## Conclusions

Seed dispersal is fundamental to the structure and function of plant communities, but its complexity and heterogeneity impede mechanistic understanding and quantitative prediction of seed dispersal processes and their disruption. Although it requires careful planning to bridge various vocabularies and epistemologies, assembling interdisciplinary working groups that leverage diverse approaches, tools and perspectives provides the potential for novel insights to emerge and new tools to bridge knowledge gaps. The diversity of disciplines, geographic regions and expertise represented in this Special Issue yield a range of perspectives and insights and, we hope, will stimulate further collaborations to advance seed dispersal ecology and conservation.

## Supporting Information

The following additional information is available in the online version of this article—

File S1. Workshop schedule.

plaa010_suppl_Supplementary_Material
