# Supplementary material for: Introduction to the Special Issue: The role of seed dispersal in plant populations: perspectives and advances in a changing world
Source: AoB Plants. 2020 Mar 13;12(2):plaa010. doi: 10.1093/aobpla/plaa010 (PMC7164217; doi:10.1093/aobpla/plaa010)
Supplement: plaa010_suppl_Supplementary_Material [file plaa010_suppl_supplementary_material.pdf]

SUPPORTING INFORMATION

Seed Dispersal Workshop Schedule
Location: National Socio-Environmental Synthesis Center, Annapolis, Maryland
Dates: May 9-13, 2016

Research Questions: During the workshop we will address the following three research questions focused on the role of dispersal in plant populations:

1. What is the relative role of different dispersal vectors (e.g., animal, wind, water, etc.) in the overall pattern of seed dispersal and recruitment?
2. To what extent does dispersal (and different dispersal vectors) affect plant population growth rate and population spread?
3. What plant and dispersal vector traits can be used to predict plant population responses to global change?

Objectives: For each research question, we will:

1. evaluate how existing empirical information, theoretical predictions, and recent analytical, computational, and statistical advances can be integrated;
2. identify remaining gaps in data, theory, or quantitative approaches; and
3. determine how such gaps can be best addressed and by whom.

Products from the overall workshop

1. Develop **common language** for collaboration during the workshop
2. **Identify framework, opportunities, and strategies** for synthesizing data with theory/math
3. Identify **data gaps**
4. **Synthesis paper #1** with a focus on uniting mathematical, theoretical, and empirical approaches to studying seed dispersal. It will summarize how each field has traditionally covered this topic, and how an integrated view provides new insight, highlighting promising new approaches, and an assessment for future directions. Would incorporate common language (product 1 above) into the paper, and the "framework for synthesis" (product 2 above). The organizing committee will send the beginnings of a framework to the workshop that participants can expand upon and work within. This will result in a synthesis manuscript.
5. **Synthesis paper #2** with a focus on understanding the effects of dispersal on plant recruitment, population growth and spread. Structured using our three research questions. Will summarize existing data/theory/math and describe data gaps (product 3 above). The organizing committee will send ideas prior to the workshop. This will result in at least one synthesis manuscript. Alternatively, we could split this into 3 papers and address each with models and data provided by participants.
6. **Working groups:** Additional working groups comprised of mathematicians, theoreticians, and empiricists will be developed during the meeting to address ideas that arise through discussion.
7. **New collaborations/future work**

Pre-Workshop Virtual Meeting with Participants: Week of April 18

1. Very quick introductions by everyone (< 10 sec - say name, where they are located). Reference the participant bios on the website.
2. Workshop coordinators will lead a discussion of overall goals for the workshop.
3. Workshop coordinators discuss the expectations for all participants prior to the workshop.

Overview of Workshop Activities

We will focus one full day on one research question. The *panel discussions* and *working groups* will aim to serve as a distillation process for ideas to include in the synthesis papers. In order to facilitate discussion and distill ideas for working groups, etc., we have broken up the beginning of each question into presentations of the background, panelists to lead discussion of gaps, and then a chance for the rest of the participants to respond. **Need facilitators who are strong and willing to tell participants to wait until after the panelists discuss to respond to presenters and panelists.**

For each question, we will have the following activities:

Presentations - **Introduction to session/research question/what is already known** regarding each research question by one of the workshop organizers + two other people (addressing empirical, theoretical, mathematical angles) as a coordinated powerpoint presentation on relevant background on the approaches of theory, math, and data and existing models and data. The two additional presenters will be selected by the workshop organizer using her knowledge of participants' research and the self-ranking of each participant's expertise on the research questions.

Panel Discussion – We will organize three panel discussions that will take place within the first three days of the workshop. Each panel discussion will focus on one of the three questions. Every participant will be assigned to a panel, except for the speakers in the above presentations.

There will be ~ 7 participants representing math, theory, and empirical approaches on each panel. Participants will be assigned to one of the three panels based on their responses to a survey asking them to rank their expertise/interest across the three research questions. Each participant will be given 2 minutes to respond to the presentation they just heard and further expand on knowledge gaps and potential approaches for addressing the research question of the day. Following a brief statement from each panelist, there will be a discussion among the panelists and presenters.

Following the panel discussion, there will be a facilitated discussion in which participants will ask questions and discuss what they've heard with panel members. As ideas are developed, they will be recorded on the whiteboard and photographed at the end of the session. The coordinators would come up with a few tangible, specific examples based on the datasets and modeling approaches we have and will include these ideas on the post-it note wall. The rubric will help with distilling ideas for working groups. This may be more about forming consensus among participants then developing rules. Coordinators will come up with themes/categories for working groups.

During this session, the 20 audience participants should also identify commonalities and differences in jargon, epistemologies, approaches, and how disciplines interact.

For the panel discussion, we will need a recorder to write ideas on the board and facilitators.

Working groups: Each group will have representatives from empirical, theoretical, and mathematical approaches. Before the workshop, coordinators will also make note of people who might have complementary skills or products (e.g., data + models) that would collaborate well together. Coordinators will make sure that some groups are addressing key questions needed for Synthesis papers #1 and #2. Each group will be asked to address each objective (integration of existing information, remaining gaps, and potential solutions for filling gaps) for the research question of that day and in the context of the particular idea/questions/case study originating from the panel session. Their task is to refine ideas from panel discussion into something manageable for a paper.

For each working group, people could have the chance to be in two working groups in case the first round of working groups doesn't go well. We will organize participants into working groups, and after about 50 minutes let participants come back together, report out briefly, and people can go back into those groups or reorganize into other working groups.

Working groups will be required to fill out a template of the rubric that coordinators can synthesize at night. This format should balance structure vs flexibility/creativity (e.g. outline, powerpoint, diagram, etc.)

Each working group should have a recorder/note-taker and a leader.

WORKSHOP SCHEDULE

8:00-10:00 MONDAY, MAY 9

Introductions

Goals: Begin group building to make sure everyone feels comfortable and has a sense of who the other participants are and generally what type of research they do. This will continue throughout the meeting with various activities.

8:00 – 8:30 *Breakfast*

- Goal: give everyone a chance to start talking and getting to know each other.

8:30 – 9:00 *Coordinators introduce workshop*

IT needs: projector, virtual participants

- Coordinators briefly introduce themselves again
- Introduce logistics & SESYNC (Haldre)
- History of this Workshop (Clare)
    - Briefly revisit goals discussed in pre-workshop meeting.
    - Focus areas: See research questions and objectives
- Products: What will we be aiming to accomplish this week and thereafter? (Noelle)
    - We anticipate at least two products including a 1) framework for synthesizing mathematical and ecological approaches to studying seed dispersal ecology and 2) the integration of quantitative and empirical approaches to address the role of seed dispersal in plant populations, highlighting promising new approaches and assessing future directions to fill identified gaps. We anticipate new collaborations among scientists, with the capacity to advance the field of dispersal ecology under global change.
    - This shared learning environment will have an outcome of a couple of publications
    - Figure of workflow and strategies – will revisit throughout the workshop
        - The repetitiveness of the workflow ensure we will achieve our goals
- Introduction to Idea Board (Haldre)
    - Goal: For participants to add research ideas for self-directed working groups (if there is time on Friday) and to start new collaborations outside of the workshop
    - Ask our trios to ask people to write down ideas on sticky notes and add to idea board and data board.
    - Guidelines for using data during the workshop

9:00-10:00 *Participant introductions (speed networking - Clare)*

IT Needs: Virtual participants

- Divide into 3 groups of 11 and rotate after 20 minutes. Mingle! Each person within a group gets 2 minutes to share: name, affiliation, interest in this workshop, general research interests and system. Each individual has with them index cards summarizing these items – pass one to each person in the group like a business card exchange.
- Organizers each in 1 room:
  - Roles: keep track of time, monitor virtual person, direct half of people moving to another location
- Goal: face-to-face communication with each participant in the workshop, breaking the ice. Each participant will walk away with a card for each person that they can reference as they continue to form relationships
- Timekeeper will enforce 20 minute time period

10:00-12:30 MONDAY, MAY 9

Introduction to other disciplines

Goals: Make sure everyone has a sense of what the big questions are in each discipline, and how each discipline addresses seed dispersal research.

How do different subdisciplines approach the study of dispersal? Are there differences in how we develop research questions? What are the benefits and difficulties of integrating approaches and interdisciplinary collaboration?

Define jargon and develop a common language to facilitate communication (product 1).

10:00-10:05 Haldre introduces flash points and idea boards
Instructions, discussion of sub-discipline differences, and discussion of perspectives, approaches, & jargon, including some initial examples on a flipchart paper

10:05 – 10:20
Each person diagrams the study of seed dispersal from their perspective to bring to the small group. Diagrams will illustrate the type of questions participants work on - not meant to be definitive but too start and frame discussions.

10:20–10:30 *Get into small groups* (one group per discipline = 3 groups)

- Goal: Each group summarizes and presents how their sub-discipline studies seed dispersal.
- Each group will illustrate and describe their approach on white boards using the individual diagrams as starting point.
- Tell participants to write big and take pictures of their work!

10:30-11:00 BREAK

11:00-11:15 *Finalize Small group discussion* (one group per discipline = 3 groups)

- Goal: Each group summarizes and presents how their subdiscipline studies seed dispersal.
- Each group will illustrate/describe their approach on white boards.
- Tell participants to write big and take pictures of their work!

Note: The following two morning sessions will be video recorded for coordinators to distill ideas for MS #1

11:15-12:00 *Groups report back, Noelle* (10 minutes per group to present, plus 5 min for questions and changing between groups)
IT NEEDS: Video recorder for organizers only; Virtual participants

- Disciplinary groups present their summary. Can use the white boards or projector.
- Clarification questions only – meat in the follow-on discussion
- Tell participants to listen carefully and take notes on interdisciplinary similarities and differences. Are there differences in what kinds of questions are approached and how? How do concepts differ? How are concepts described? Does terminology differ? How is dispersal approached? Note down any differences you notice among disciplines, not just differences in concepts. These notes will be used in the next exercise.

12:00-12:30 *Facilitated Whole group discussion, Clare facilitates– Haldre + Noelle record.*
IT Needs: Video recorder for organizers only; Virtual participants; Dedicated white board for the week

- Goal: To introduce the need for a common language.
- Discuss how disciplines differ in how they address dispersal. This includes differences in jargon, research questions, assumptions, theory, etc. We will identify/define any terms that may be used differently in each discipline and other differences in research approaches. Dedicate a white board that participants can continue to add to throughout the week. This will be summarized and revisited on Friday.
- What did participants (dis)agree on? Linked to individual or discipline? Look for unexpected
- The results of this exercise will be used in manuscript #1 and will be useful for ensuring good communication throughout the week.

12:30 – 1:30 Lunch

1:30 – 5:00 MONDAY, MAY 9

Research Question 1: What is the relative role of different dispersal vectors (e.g., animal, wind, water, etc.) in the overall pattern of seed dispersal and recruitment?

Goals: Address objectives 1-3 for this question

Leaders: Clare Aslan + 2 participants from theory and math

Choose a Designated Timekeeper
1:30-2:00 *Presentation on what is known* by leaders
IT NEEDS: Stream live and record for youtube (though don't make it public)

2:00 – 3:00 *Panel Discussion on gaps and future directions*– (7 other participants who selected this question)
IT NEEDS: Video record for organizers; Virtual participants
Facilitator: Clare
Recorders: Haldre and Noelle
Panelists (1 virtual participant)

Math, Theory, Empirical
- 2:00 – 2:30 Each panel member has 2 minutes to reflect on presentation and expand on outstanding gaps, questions, & approaches
  Record identified gaps and questions on whiteboard for use in working group formation
- 2:30 – 2:50 Facilitated discussion on additional gaps and questions
- 2:50 – 3:00 Organization and Distillation for working groups

3:00 – 3:30 *Afternoon break.*

During break:
- 3:00-3:10 Participants sign up for their top 3-5 knowledge gaps/approaches they are most interested in addressing during working groups – identified during the facilitated discussion.
- 3:10-3:30 Coordinators develop working groups based on signups.

3:30-5:00 *Working group R1*

Each group decides on presenter and recorder
Facilitator doesn't participate
Written report back to organizers.

5:00 – 6:00 Happy Hour (Could also be wrapping up the conversations of the day while happy hour is beginning – so a little time slop is okay here)

6:30 Working dinner* with group
*Workshop organizers: Ideas and questions emerging from these sessions will be recorded during the day and synthesized by workshop organizers in the evening. Ideas relevant to each research question will be pulled out for use during the discussions in Days 4-5.

9:00-12:30 TUESDAY, MAY 10

Goals: Research Question 1, continued

Breakfast at hotel: Clare and Haldre use this time to informally check-in with participants and get informal feedback of how the working groups are progressing

Choose a Designated Timekeeper

9:00 – 9:20 Name game/ice breaker
Pass around Justin Timberlake cardboard cut-out

IT NEEDS: Virtual participants

9:20 – 10:00 Check-in

- Coordinators go over any meeting announcements.
- Working groups give 5 minute update - summarize outcomes from Day 1 including how current working groups and additional research questions that have been identified would fit objectives for Q1.
- Participants decide whether they want to continue in this group or join new group.

10:00 – 10:30 *Working groups R1*
Participants assemble into working groups (continued from before or new groups). Leader from each working group reminds group what their group will be doing, and then participants can stay in same working group or join a new working group. Continue developing ideas.

10:30-11:00 BREAK

11:00 – 12:00 *Working groups R1*

- Fill out template before lightening talks

12:00 - 12:30 *Lightning talks*
IT Needs: Virtual participants

- Working groups report back to the entire group. Each group gets 5 minutes.
- What worked and what didn't? What did you find? What gets added to the ideas board for Friday discussions?
- 5 minutes per working group
- 10 minutes for epistemology. Revisit white board of epistemologies
- Near final product to be given to coordinators for integration into Synthesis MS #2

12:30-1:15 LUNCH

1:15 – 1:30 GROUP DISCUSSION – FEEDBACK

- Get feedback from group about the workshop structure. What worked? What didn't work? How do you like the transitions from panel discussion to working groups?

1:30-5:30 TUESDAY, MAY 10

Research Question 2: To what extent does dispersal (and different dispersal vectors) affect plant population growth rate and population spread?

Goals: Address objectives 1-3 for this question

Leaders: Noelle + 2 participants from math and empirical ecology

1:30-2:00 *Presentation on what is known* by leaders
Location: Blue room
IT NEEDS: Stream live and record for youtube (though maybe don't make it public)

2:00 – 3:00 *Panel Discussion gaps and future directions* – (7 other participants who selected this question)
IT NEEDS: Video Record for organizers; Virtual Participants
Facilitators: Haldre and Clare
Panelists (includes 1 virtual participants)

Math, theory, empirical

2:00 – 2:30 Each panel member has 2 minutes to reflect on presentation and expand on outstanding gaps, questions, & approaches
Record identified gaps and questions on whiteboard for use in working group formation

2:30 – 3:00 Facilitated discussion on additional gaps and questions

3:00 – 3:30 *Afternoon break.*

During break:

3:00-3:10 Participants sign up for their top 3 knowledge gaps/approaches they are most interested in addressing during working groups – identified during the facilitated discussion.

3:10-3:30 Coordinators develop working groups based on signups.

3:30-5:00 *Working group R2*
Locations: Fishbowl, Cave, Blue room, Open space – the first 3 will be set-up for virtual participation.

5:00 Group walks to water

6:30 Working dinners* in small groups

*Workshop Organizers: Ideas and questions emerging from these sessions will be recorded during the day and synthesized by workshop organizers in the evening. Ideas relevant to each research question will be pulled out for use during the discussions in Days 4-5.

9:00-12:30 WEDNESDAY, MAY 11

Research Question 2: To what extent does dispersal (and different dispersal vectors) affect plant population growth rate and population spread?

Goals: Address objectives 1-3 for this question

Breakfast at hotel: Clare and Haldre use this time to informally check-in with participants and get informal feedback of how the working groups are progressing

Choose a Designated Timekeeper
9:00 – 9:20 Ice breaker/check-in
IT NEEDS: Virtual participants

9:20 – 10:00 Check-in
- Coordinators go over any meeting announcements.
- Working groups give 5 minute update - summarize outcomes from Day 2 including how current working groups and additional research questions that have been identified would fit objectives for Q2.
- Participants decide whether they want to continue in this group or join new group.

10:00 – 10:30 *Working groups R2*
Participants assemble into working groups (continued from before or new groups). Leader from each working group reminds group what their group will be doing, and then participants can stay in same working group or join a new working group. Continue developing ideas.

10:30-11:00 BREAK

11:00 – 12:00 *Working groups R2*
- Fill out template before lightening talks

12:00 - 12:30 *Lightning talks*
Location: Blue room
IT NEEDS: Virtual participants

- Working groups report back to the entire group. Each group gets 5 minutes.
- What worked and what didn't? What did you find?
- 5 minutes per working group
- 10 minutes for epistemology. Revisit white board of epistemologies
- Near final product to be given to coordinators for integration into MS

12:30-1:30 LUNCH

1:30-5:00 WEDNESDAY, MAY 11

Research Question 3: What plant and dispersal vector traits can be used to predict plant population responses to global change?

Objectives: Address objectives 1-3 for this question

Leaders: Haldre + 2 participants from theory and math

1:30-2:00 *Presentation on what is known* by leaders
Location: Blue room
IT NEEDS: Stream live and record for youtube (though don't make it public)

2:00 – 3:00 *Panel Discussion on gaps and future directions* – (7 other participants who selected this question)
IT NEEDS: Video record for organizers
Panel (1 virtual participants)
Math, Theory, Empirical
Facilitators: Clare and Noelle
2:00 – 2:30 Each panel member has 2 minutes to reflect on presentation and expand on outstanding gaps, questions, & approaches
Record identified gaps and questions on whiteboard for use in working group formation
2:30 – 3:00 Facilitated discussion on additional gaps and questions

3:00 – 3:30 *Afternoon break.*

During break:
3:00-3:10 Participants sign up for their top 3 knowledge gaps/approaches they are most interested in addressing during working groups – identified during the facilitated discussion.
3:10-3:30 Coordinators develop working groups based on signups.

3:30-5:00 *Working group R3*

6: 30 Working dinners* in small groups

*Workshop Organizers:

- Ideas and questions emerging from these sessions will be recorded during the day and synthesized by workshop organizers in the evening. Ideas relevant to each research question will be pulled out for use during the discussions in Days 4-5.

- Revisit workshop schedule and structure for Thursday and Friday and the participants that represent each question to make sure it still makes sense or whether the schedule or groups need to be revised.

9:00-12:30 THURSDAY, MAY 12

Research Question 3: What plant and dispersal vector traits can be used to predict plant population responses to global change?

Objectives: Address objectives 1-3 for this question

9:00 – 9:30 Check-in

IT NEEDS: Virtual participants

- Coordinators go over check-out and ground transportation procedures.
- Working groups give 3 minute update - summarize outcomes from Day 3 including how current working groups and additional research questions that have been identified would fit objectives for Q3. Note that the goal is to id knowledge gaps, not necessarily to have every working group lead to a paper.
- Participants decide whether they want to continue in this group or join new group.

9:30 – 11:30 *Working groups R3*

.

- Participants assemble into working groups (continued from before or new groups). Leader from each working group reminds group what their group will be doing, and then participants can stay in same working group or join a new working group. Continue developing ideas.

11:30 - 12:00 *Lightning talks*

IT Needs: Virtual participants

- Working groups report back to the entire group. Each group gets 5 minutes.
- What worked and what didn't? What did you find?
- 5 minutes per working group
- Near final product to be given to coordinators for integration into MS

12:00-12:30 Themes – brainstorming (what have we seen repeatedly?)

IT Needs: Virtual participants

Leader: Clare

12:30-1:30 LUNCH

1:30-3:00 Walk to water and ice cream

3:00-6:00 THURSDAY, MAY 12

Wrapping up all three questions and all working groups

3:00-3:30 Working groups Q1

- Participants assemble into the groups for Q1. Answer the following questions: a) will this working group continue in any form; b) who will coordinate the continuation; c) whether or not it continues, describe the knowledge gaps and novel focus areas of this working group by completely filling out the template and cleaning it up.
- If group will continue, succinctly describe the question, knowledge gap, and approach in a paragraph, and we will post it on the board for people to read over/think about/decide if they want to participate.
- If two working groups are similar, they might consider merging if a combined group would be more likely to move an idea forward.

3:00-3:30 Working groups Q2

3:30-4:00 Working groups Q3

4-4:30: Working groups that will be continuing will report back and invite collaborators. One question at a time, the coordinator for each question describes the question/approach/plan for moving forward, and posts the paragraph on the board.

4:30-6:00 Happy hour

- Ideas board discussions, people sign up for groups they want to continue with
- Participants gather in groups to discuss Idea Board and participant-led ideas. Feel free to meet outside at tables if weather permits.

6:30 Working dinner with group

8:00 Shuttle to hotel

9:00-12:00 FRIDAY, MAY 13

Wrapping up workshop, discussion of next steps

9:00 – 9:15 Revisit why we are here: to identify knowledge gaps and network.

"Ideas paper" emerging from this workshop (integrate results from Themes discussion and direct participants to outline on Notebook) (**Leader**: Noelle)

Identify the single knowledge gap (10 minutes; small sticky)

9:15 – 10:00 Discussion on interdisciplinary process and common themes (**Leader**: Clare)

10:00 – 10:30 Disciplinary groups reassemble

- Discuss outcomes of workshop
- How did workshop advance disciplinary understanding of seed dispersal? What new ideas did it stimulate?

10:30-10:45 Report out on disciplinary advances (organizers record)

10:45-11:00 BREAK – participants with a desire to assemble to discuss other ideas (disciplinary or interdisciplinary), write those ideas on the board

11:00 -12:30 Coordinators facilitate discussion on ways forward (**Leader**: Haldre)

- Data needs
- Is seed dispersal important? Discussion on raising social profile of seed dispersal
- Potential next steps and collaborative points
    - CoDisperse Network
    - Idea board
    - Working group sign-ups
- What have we have applied for and what else should we apply for?
- Remind everyone to fill out electronic survey for additional feedback

12:30 – 1:30 Lunch

- Thank you!

1:30 – 5:00 Participants depart after lunch. Organizers will lead synthesis of workshop for resulting manuscript.

5 pm- everyone departs
